# 144. Impact of Rapid Identification and Resistance Gene Detection using an Algorithm-Based Approach in Gram-Negative Bacteremia

**DOI:** 10.1093/ofid/ofad500.217

**Published:** 2023-11-27

**Authors:** Jasanjeet Jawanda, Lloyd Clarke, Hannah M Creager, Ryan K Shields

**Affiliations:** University of Pittsburgh Medical Center, Vincennes, Indiana; Antibiotic Management Program, UPMC Presbyterian Hospital, Pittsburgh, PA, Pittsburgh, Pennsylvania; University of Pittsburgh Medical Center, Vincennes, Indiana; University of Pittsburgh, Pittsburgh, PA

## Abstract

**Background:**

Time to appropriate antimicrobial therapy is the most important predictor of survival among patients with sepsis due to Gram-negative bacteria (GNB). Infections due to multidrug-resistant (MDR) pathogens often result in inappropriate empiric treatment (tx). The GenMark ePlex identifies organisms and resistance markers within 1.5 hours from blood culture positivity compared to 48-72 hours by conventional methods.

**Methods:**

We are conducting a single-center study to assess outcomes before and after ePlex implementation. The pre-intervention period included patients with GN bacteremia from January to June 2021. Patients were excluded if they received comfort-only care within 48 hours, had polymicrobial GN bacteremia, or were infected by an organism not on the ePlex panel. Primary outcome is time to in-vitro active therapy from blood culture collection.

**Results:**

208 patients were identified during the pre-intervention period; 154 met inclusion criteria. Median age was 64 years, 49% were male, and 28% were immunocompromised. Median (IQR) Charlson Comorbidity index and Pitt Bacteremia scores were 6 (4-8) and 2 (1-8), respectively (**Table 1**). 16% of patients were infected by MDR GNB and 21% received inactive empiric tx. Overall median (IQR) time to in-vitro active tx was 3.9 (1–19) hours, but was 39.5 (13.6-71.9) hours among those who received inactive empiric tx. Median (IQR) time to first antibiotic modification was 2.7 (1.0-3.4) days. 38% of patients were transitioned to oral antibiotics at a median (IQR) of 4.2 (3.1-6.1) days. The median (IQR) duration of tx was 15 (10-17) days; 4.5% of patients were treated with ≤ 7 days. Median (IQR) length of hospitalization was 15.5 (7-36) days. 7.8% were re-admitted within 30 days (**Table 2**). In-hospital and 30-day mortality rates were numerically higher among patients who received inactive empiric therapy (27% and 21%, respectively) when compared to patients who received active empiric therapy (16.5% and 12%, respectively) (**Figure 1**).

Results Table 1: Patient demographics, underlying conditions and infection and treatment characteristics in the pre-intervention group

**Figure:**
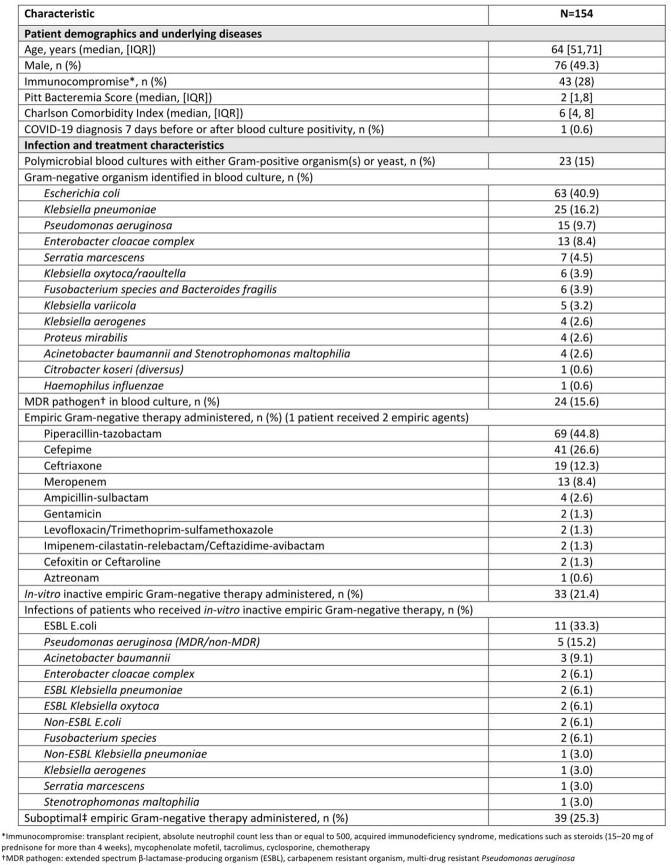

Results Table 2

**Figure:**
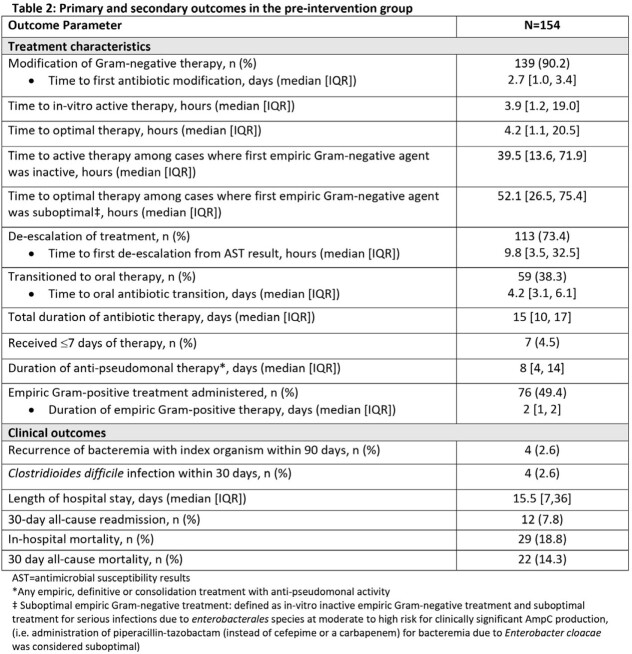

Results Figure 1

**Figure:**
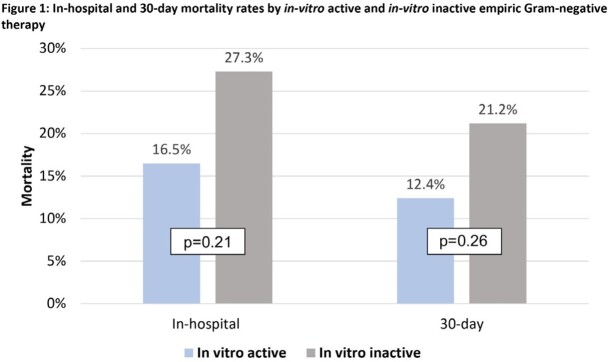

**Conclusion:**

Our pre-intervention data highlight opportunities to improve the management of GN bacteremia. Implementation of ePlex is likely to decrease the proportion of patients treated with inactive therapy, shorten time to optimal tx and reduce lengths of stay.

**Disclosures:**

**Ryan K. Shields, PharmD, MS**, Allergan: Advisor/Consultant|Cidara: Advisor/Consultant|Entasis: Advisor/Consultant|GSK: Advisor/Consultant|Melinta: Advisor/Consultant|Melinta: Grant/Research Support|Menarini: Advisor/Consultant|Merck: Advisor/Consultant|Merck: Grant/Research Support|Pfizer: Advisor/Consultant|Roche: Grant/Research Support|Shionogi: Advisor/Consultant|Shionogi: Grant/Research Support|Utility: Advisor/Consultant|Venatorx: Advisor/Consultant|Venatorx: Grant/Research Support

